# Correspondence

**Published:** 1860-03

**Authors:** J. S. Lindsey

**Affiliations:** Frederick City


### Correspondence.

Messrs. Editors:—For a number of years I've noticed many different accounts of hemorrhage and many different modes of treatment. In the last *Dental Journal* there is a letter from Dr. Harvey, which confirms me in my plan of treatment in professional cases of hemorrhage—an account of which I will give you and which you can insert in the *Register*, if you think it will benefit your readers

In 1855 I extracted for a lady the second bicuspid on left superior maxillary, which at the time passed off without any appearance of hemorrhage. All continued well for five days, at which time hemorrhage commenced and was suffered to continue for sixteen or eighteen hours before sending for assistance. On my arrival I found no time was to be lost, as a few hours would terminate her existence. I immediately applied a pill made of gum myrrh and tannin, made very stiff and rolled out to correspond in shape to the root of the tooth and forcing it into the orifice as tightly as possible, placing a pledget of cotton against it, directing the patient to close the mouth to keep it in place, which stopped the flow immediately. I am fully convinced profused cases of hemorrhage, from extracting teeth, must be stopped by pressure. I prefer the gum myrrh or gum kimo, as they will gradually dissolve and pass out—giving no trouble to remove. Believing this course to be the safest and most reliable, you may, if you think proper, give it a place in the *Register.*

Yours, truly, J. S. LINDSEY.

*Frederick City*, Feb. 13, 1860.

---

### NOMENCLATURE OF DENTAL CARIES.

Messrs Taft and Watt, *Gentlemen*:—The action of *acids* has made dental caries *so* simple a pathological phenomenon, that authors now esteem all classification of the *thing* called caries as "unnecessary" incumbrances or meaningless epithets. "*Deep-seated, superficial,*" etc., etc., are all considered useless "appellations" by "Harris," who uses them no doubt, as he does the term "caries" itself, because "we know of no better names."

It would be a convenience, if no more, to have the "different stages" of caries *mapped out* and appropriately nomenclatured. In medicine this is the case in all local diseases. But in dentistry there is a meagerness in terms that is very often seriously felt. We have thought the *poverty* of our nomenclature of *caries of the teeth* should be removed by the *coining* of such terms as would be in accordance with the progress of the science. Some names have suggested themselves to our mind which we do not remember to have seen used or applied to designate the degrees or stages of this affection. They have some appropriateness; but whether they shall prove acceptable to the professional minds, is another question. No harm can result from suggesting them; and with your permission, we hereby present them to the consideration of our brethren.

We find medical names of local diseases are often derived from the names of the parts affected. For instance, *laryngitis*, or inflammation of the larynx, is termed *mucous* larygitis, when the mildest form of the disease exists; and it is called *sub-mucous* laryngitis (the form of which Washington died,) when the inflammation is of the highest grade, and the subjacent areolar tissue is involved.

Caries attacks or is found to exist in the *enamel* and the dentine. Taking these as the basis of a nomenclature for caries, the following *prefixes* will constitute words suggestive of the locality and stages of the affection. Caries is found existing *on* and *in* the body of the enamel and dentine.

First, then, *Epienamel* decay. This is caries of the simplest form existing, as the name indicates, on the external portion of the enamel. Second, *Intraenamel* decay. This would express a decay advanced *within* the substance of the enamel. Third, *Epidentinal* decay. This would designate caries limited to the external portions of the dentine. Fourth, *Intradentinal* decay. This would be the name for caries "deep-seated," or involving the *internal* parts of the dentinal tissue.

It is respectfully submitted, whether the above nomenclature be not more definite and significant than the terms, "*superficial*" and "*deep-seated*," which are the only "appellations" *we* "know of" whereby we "designate the different stages of the same disease?"

The terms *epienamel*, *infradentinal*, and so forth, fix definately the stages of caries as it advances from tissue to tissue, or from the one part of the tissue to another part. It will be serviceable in diagnosing caries, causing the dentist to investigate the actual extent of the affection. The pathological phenomena presented or existing in an *epidentinal* form of caries might be different from those to be found when caries should be *infradentinal*. A soft texture invites the attack of caries and facilitates its triumphant progress, while a solid normal texture of the enamel and dentine offers a strong resistance, and struggles long with the "enemy!" The former structural condition would evidently pre-dispose to the speedy development of *infradentinal* caries—the whole body of the tissue becoming, at times, disorganized before the existence of the "insidious" disease becomes known! But the latter structural condition, namely, that of a solid or perfect description, would present generally decay in an *epienamel* or *epidentinal* form, in consequence of the resisting power inherent in the tissues attacked.

When decay is epienamel or epidentinal, and the texture of tissue is good or resistant, the operation of filing for these forms of caries would be, in the main, *safe* and "judicious." But when the structure is weak, soft, susceptable, offering little or no resistance to *carial* attacks, then even these mild forms of the disease would demand a different treatment—*perhaps, timely filling;* with a good deal of *nursing, afterward!*

A few days ago (10th instant,) we treated two central incisors for a lady *æt* 28. *Caries* existed on the mesial approximate sides. Enamel and dentine of good texture. *Left central;* decay existing only *epidentinally.* The resisting power of the tissue kept the disease partly at check. The two incisors touching, we filed away the entire *epidentinal decay*, leaving the tooth in a safe condition. The right central was affected to a greater degree. The caries was *infradentinal.* The affection traversed the epidentinal regions of the tissue, more than was deemed prudent to attempt to remove by *filing.* The tissue was penetrated two or more lines, at the greatest depths of the cavity. Had earlier application been made, this tooth would have been "a fit subject for the file;" but the disease had progressed too far. This tooth we filled with *gold.* Yours,

GEO. S. FOUKE.

*Westminster, Md.*, Feb. 13, 1860.

